# The Effectiveness and Safety of Endoscopic Intermuscular Dissection for the Treatment of Rectal Gastrointestinal Stromal Tumors: A Pilot Feasibility Study

**DOI:** 10.3390/jcm15145712

**Published:** 2026-07-21

**Authors:** Seong-Jung Kim, Eun Jeong Kim, Min Hyeok Lee, Jun Lee

**Affiliations:** Department of Internal Medicine, Chosun University College of Medicine, 309 Pilmun-daero, Dong-gu, Jeonnam-Gwangju Special Metropolitan City 61452, Republic of Korea; ygegh@chosun.ac.kr (S.-J.K.); illusion1211@naver.com (E.J.K.); minhyg123@gmail.com (M.H.L.)

**Keywords:** gastrointestinal stromal tumor, endoscopy, rectum

## Abstract

**Background/Objectives:** Rectal gastrointestinal stromal tumors (GISTs) near the dentate line (DL) present significant surgical challenges due to anatomical constraints and difficulty preserving anal function. Endoscopic intermuscular dissection (EID) is a viable alternative in these cases. **Methods:** We retrospectively reviewed the medical records of patients who underwent EIDs for rectal GISTs near the DL between January 2015 and December 2023. The primary outcomes assessed were procedural efficacy—measured by en bloc and complete resection rates—and safety, evaluated regarding delayed bleeding, perforation, and tumor recurrence. **Results:** In total, 10 patients underwent rectal GIST EIDs. The mean tumor size, procedure time, and resection speed were 25.70 ± 14.09 mm, 81.30 ± 46.20 min, and 16.91 ± 9.94 mm^2^/min, respectively. The en bloc and complete resection rates were 100% and 70%, respectively. Compared with endoscopic submucosal dissection (ESD) for rectal epithelial lesions, there were no significant differences in procedure times (81.30 ± 46.20 min vs. 76.50 ± 32.42 min, *p* = 0.758) or complete resection rates (70% vs. 95%, *p* = 0.095). Regarding safety, delayed bleeding (10% vs. 10%, *p* = 1.000) and perforation (10% vs. 0%, *p* = 0.333) rates did not differ significantly between the two groups. During a median 36-month follow-up, one recurrence occurred in a patient with a high-risk GIST. **Conclusions:** EID for rectal GISTs near the DL appears to be a feasible and potentially safe treatment option in carefully selected patients. It may represent a less invasive alternative to surgery, particularly when preservation of anal function is a priority. Nevertheless, validation in larger prospective studies is required.

## 1. Introduction

Rectal gastrointestinal stromal tumors (GISTs) are rare but clinically significant due to their malignant potential and complex management. When located near the DL, these tumors present unique challenges because achieving complete resection often comes at the cost of compromising anal function. The distal rectum’s anatomical constraints render standard surgical approaches high-risk. Consequently, these procedures are frequently associated with substantial morbidity, including sphincter dysfunction and the potential need for permanent colostomies.

Neoadjuvant therapy with imatinib has been used to reduce tumor sizes before surgery, with the goal of enhancing resectability and preserving critical structures [1]. However, its effectiveness in rectal GISTs remains poorly validated, and evidence regarding its impact on long-term outcomes is limited.

Endoscopic intermuscular dissection (EID) has emerged as a minimally invasive and organ-preserving alternative for managing subepithelial lesions, including GISTs. EID enables en bloc resection with a potential for curative intent, while minimizing the risk to anorectal function [2,3,4,5,6,7]. Although several case studies have reported the feasibility of EIDs for rectal submucosal lesions, data specifically addressing rectal GISTs near the DL are scarce.

Therefore, this study aimed to evaluate the feasibility, safety, and long-term outcomes of EIDs for rectal GISTs near the DL. We compared EID outcomes for rectal GISTs with endoscopic submucosal dissection (ESD) outcomes for epithelial lesions in the same anatomical region to further assess EID’s utility and safety.

## 2. Materials and Methods

### 2.1. Study Design and Patients

**Figure 1:**
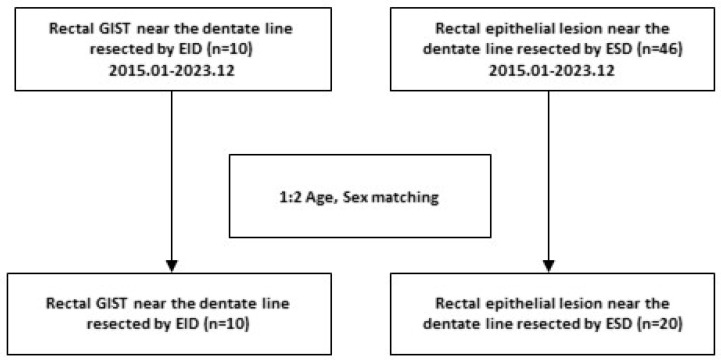
Flow chart of this study.

We retrospectively collected and analyzed clinical, endoscopic, and histopathological data from patients who underwent EIDs for rectal GIST at Chosun University Hospital between 1 January 2015, and 31 December 2023. Upfront EID was offered to patients who declined surgical resection because of concerns regarding permanent colostomy and whose lesions were considered amenable to endoscopic resection based on an endophytic growth pattern, in the absence of adjacent organ invasion or distant metastasis. During the same period, ESDs were performed on 42 patients with rectal epithelial lesions near the DL. From this group, 20 patients were selected as the comparison cohort using 1:2 age and sex matching with the rectal GIST group (Figure 1).

The primary objective of this study was to evaluate the efficacy and safety of EID for rectal GISTs. The secondary objective was to compare these results with ESD for rectal epithelial lesions in the same anatomical location and assess whether EID is a viable and safe treatment strategy for rectal GISTs in this challenging region.

This study was conducted in accordance with the principles of the Declaration of Helsinki and approved by the Institutional Review Board of Chosun University Hospital (approval no. CHOSUN 2024-10-037-001). This study was reported in accordance with the Strengthening the Reporting of Observational Studies in Epidemiology (STROBE) statement.

### 2.2. EID Procedures

**Figure 2:**
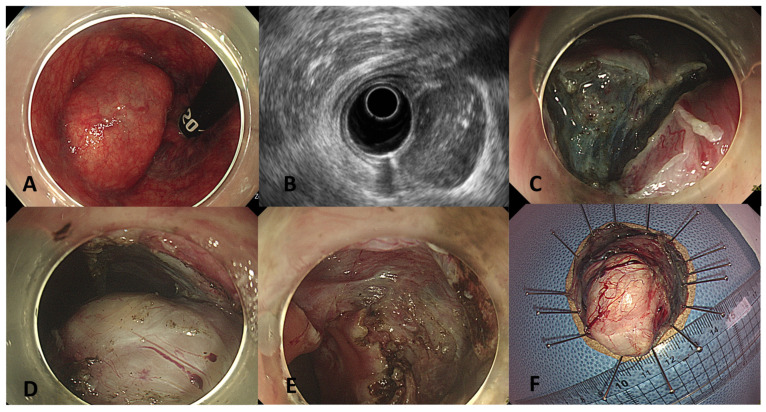
Rectal gastrointestinal stromal tumors near the dentate line resected en bloc by endoscopic intermuscular dissection (Supplementary Materials). (**A**) Endoscopic view showing a subepithelial tumor located adjacent to the dentate line. (**B**) Endoscopic ultraso-nography demonstrating a hypoechoic lesion originating from the muscularis propria. (**C**) Mucosal incision and initiation of intermuscular dissection after circumferential marking. (**D**) Exposure of the tumor during intermuscu-lar dissection with preservation of the outer longitudinal muscle layer. (**E**) Post-resection defect after en bloc re-moval of the tumor. (**F**) Gross specimen after pinning, showing the completely resected tumor.

EID was performed for subepithelial tumors originating from the proper muscle layer as identified on endoscopic ultrasound (EUS). All EID procedures were performed by two experienced endoscopists under conscious sedation. Because the lesions were close to the anus, which may cause pain during the procedure, combination sedation was applied using an initial dose of 2 mg of midazolam followed by titrated doses of 20 mg of propofol as needed [8]. A single-channel, water-jet endoscope (GIF-260, GIF-HQ290, CF-H260AI, or CF-H290AI; Olympus, Tokyo, Japan), fitted with a transparent distal hood (standard or small-caliber-tip transparent hood; Olympus), was used for the procedures. Carbon dioxide insufflation was used throughout. For the submucosal injection, a solution of hypertonic saline (a mixture of 5% glycerin, 0.9% saline, and 0.008% indigo carmine) or hyaluronic acid was used. Mucosal incision and submucosal dissection were performed using one or two types of knives: Dual Knife (KD-650Q; Olympus) and Clutch Cutter (Fujifilm Co.). Incision and dissection were performed using the Endocut Q mode (effect 3, duration 2, interval 3), while the swift coagulation mode (effect 3, 40 W) was applied for dissection, and the soft coagulation mode was used for hemostasis. Initial submucosal lifting was achieved via injection, and additional injections were administered as necessary. Bleeding and exposed vessels were promptly controlled using hemostatic forceps (FD-411QR; Olympus) (Figure 2). The mucosal and intermuscular defects were not routinely closed after EID. Clip closure was performed selectively at the operator’s discretion when deep muscular injury was identified or when the risk of perforation was considered clinically significant during the procedure, in which case partial clipping of the dissected area was applied.

### 2.3. Management of Patients Undergoing EIDs for Rectal GISTs

Patients were admitted one day before the procedure, and EIDs were performed the following day. In the absence of complications, oral water intake was initiated four hours post-procedure, followed by a liquid diet in the evening. Patients were typically discharged on the day after the procedure [9]. Prophylactic antibiotics were not routinely administered. However, in cases of suspected complications, laboratory tests, abdominal radiography, and computed tomography (CT) scans were performed for further evaluation. Post-resection surveillance included a colonoscopy performed 6 months after the procedure, followed by another colonoscopy performed in the third year. CT scans were performed annually.

### 2.4. Data Collection and Definitions

We collected patient demographic data, including age, sex, body mass index (BMI), smoking status, comorbidities, and American Society of Anesthesiologists (ASA) classification. Procedure-related and histological data included bowel preparation quality, tumor size, and histopathological findings.

To evaluate the efficacy of EID, we assessed the en bloc resection rate, complete resection rate, procedure time, and resection speed. Safety outcomes included procedure-related complications, fasting duration, length of hospital stay, recurrence, and cancer-related mortality.

En bloc resection was defined as the resection of the lesion in a single piece. Complete resection was defined histologically as tumor-free vertical and lateral margins; however, because thermal cautery may affect margin assessment, pathological R1 status after EID does not necessarily indicate residual disease. The procedure time was measured from the initiation of submucosal injection to the completion of dissection. Resection speed was calculated by dividing the resected area by the procedure time (mm^2^/min). Delayed bleeding or perforation was defined as any event that occurred within 30 days following EID. Recurrence was defined as the detection of local, nodal, or distant metastases during surveillance. Risk stratification was performed according to the modified National Institutes of Health (Joensuu) consensus criteria, which incorporate tumor size, mitotic count, tumor site, and tumor rupture. As all lesions were located in the rectum (a non-gastric site), non-gastric tumors of >2–5 cm with >5/50 HPF or 5–10 cm with ≤5/50 HPF were classified as high risk, in accordance with the site-specific modification of these criteria [10].

### 2.5. Statistical Analyses

Categorical variables are presented as frequencies and percentages, and comparisons between groups were made using the chi-squared or Fisher’s exact tests, as appropriate. Continuous variables are reported as means ± standard deviations or medians with ranges, as appropriate, and compared using the Mann–Whitney U test. Kaplan–Meier analysis was performed to evaluate recurrence-free survival for long-term outcomes. Statistical significance was defined as a two-sided *p*-value < 0.05. All statistical analyses were conducted using the SPSS software (version 27.0; IBM Corp., Armonk, NY, USA).

## 3. Results

### 3.1. Clinicoendoscopic Characteristics of Patients with Rectal GISTs near the DL

In total, 10 patients underwent EIDs for rectal GISTs near the DL. The median age was 53 (20–71) years, and four patients (40%) were male. The mean BMI was 26.5 ± 3.71 kg/m^2^. Comorbidities included hypertension in three patients (30%), diabetes mellitus in one patient (10%), and one patient (10%) had coronary artery disease and was taking antithrombotic agents. All patients were classified as ASA class 2 or lower. Adequate bowel preparation was achieved in all patients.

The mean tumor size was 25.70 ± 14.09 mm and the mean procedure time was 81.30 ± 46.20 min. According to the histopathological evaluation, four lesions (40%) were classified as very low risk, five (50%) as low risk, and one (10%) as high risk (Table 1).

### 3.2. Efficacy and Safety of EIDs for Rectal GISTs near the DL

In the present study, the en bloc resection rate for EIDs of rectal GISTs near the DL was 100%, whereas the complete resection rate was 70%. Although all three incompletely resected GISTs had positive vertical margins, no relapse was observed in these cases, and recurrence occurred only in the patient with a high-risk GIST. The mean EID resection speed was 16.91 ± 9.94 mm^2^/min. Regarding safety outcomes, one patient (10%) developed delayed bleeding, which was managed conservatively. Another patient (10%) experienced delayed perforation, with a small amount of free air observed in the pelvic cavity on CT. The patient was managed conservatively without surgical intervention, and the condition resolved without complications. Post-endoscopic coagulation syndrome occurred in one patient (10%) and resolved without further complications. The median fasting duration was one day, and the median hospital stay was three days. Furthermore, no patients developed persistent anorectal symptoms, including fecal incontinence, persistent anal pain, urgency, or clinically meaningful deterioration in anorectal function during follow-up (Table 2).

Post-resection surveillance generally included a colonoscopy performed 6 months after the procedure, followed by another colonoscopy performed in the third year. CT scans were performed annually. All three patients with R1 resection lacked high-risk features and therefore did not receive adjuvant imatinib therapy. They were followed with contrast-enhanced CT every 6 months and surveillance colonoscopy at 1 year and every 3 years thereafter. During a median follow-up of 36 months (range, 20–66 months), a single case (10%) of local recurrence was reported in the patient with a high-risk GIST. No GIST-related mortalities were observed during the follow-up period. The single patient with a high-risk GIST received adjuvant imatinib following EID. Imatinib was discontinued because of a treatment-related adverse event (drug-induced pneumonitis) and was switched to sunitinib, which was subsequently discontinued owing to drug-induced hand–foot syndrome. Local recurrence at the resection site was confirmed by CT and EUS 34 weeks after cessation of systemic therapy, corresponding to 61 months after the initial EID. Surgical resection was recommended but declined by the patient; ripretinib was therefore initiated following multidisciplinary discussion.

A summary table was compiled to present the clinical characteristics, therapeutic outcomes, and prognoses of all patients with rectal GISTs treated with EID (Table 3).

### 3.3. Comparison with ESDs for Rectal Epithelial Lesions near the DL

To assess the feasibility and safety of EIDs for rectal GISTs near the DL, outcomes were compared with those of a control group of 20 patients who underwent ESDs for rectal epithelial lesions located in the same anatomical region. The control group was selected using 1:2 age and sex matching.

No significant differences were observed in the clinicoendoscopic characteristics between the two groups, including tumor size (25.70 ± 14.09 mm vs. 22.80 ± 12.46 mm, *p* = 0.551) and procedure time (81.30 ± 46.20 min vs. 76.50 ± 32.42 min, *p* = 0.758). Regarding efficacy, there were no significant differences between the GIST group and the epithelial lesion group regarding en bloc resection rate (100% vs. 100%, *p* = 1.000), complete resection rate (70% vs. 95%, *p* = 0.095), or resection speed (16.91 ± 9.94 mm^2^/min vs. 12.98 ± 11.95 mm^2^/min, *p* = 0.169). Regarding safety, delayed bleeding (10% vs. 10%, *p* = 1.000), perforation (10% vs. 0%, *p* = 0.333), and post-endoscopic coagulation syndrome (10% vs. 20%, *p* = 0.640) did not differ significantly between the two groups, although the low event rate limited the statistical power to detect such differences.

**Figure 3:**
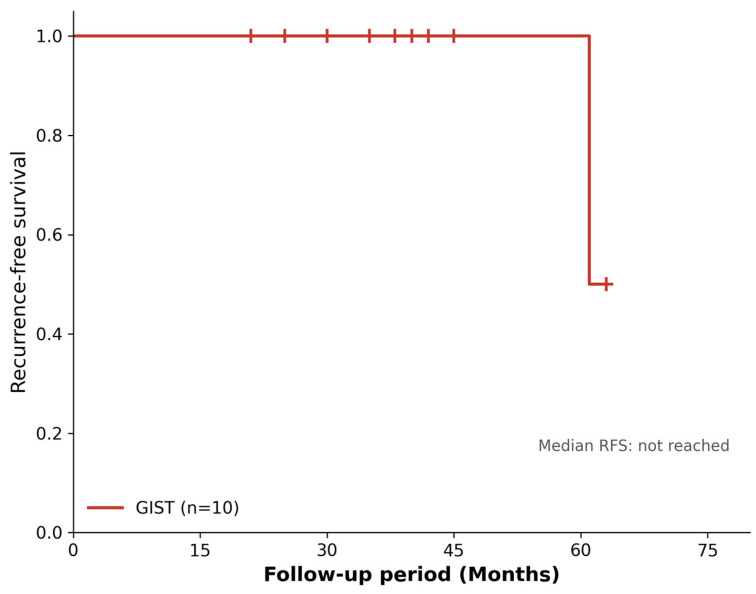
Kaplan–Meier curves for recurrence-free survival in rectal gastrointestinal stromal tumors near the dentate line.

During a median follow-up period of approximately 36 months, one case of recurrence was observed in the GIST group (Figure 3). However, no statistically significant differences were observed in recurrence rates between the two groups (*p* = 0.333). No lesion- or procedure-related mortality was observed in either group (Table 4).

## 4. Discussion

In this study, EID for rectal GISTs near the DL demonstrated favorable technical feasibility and an acceptable safety profile, with a 100% en bloc resection rate, a 70% complete resection rate, and no fatal complications. All adverse events were successfully managed with conservative treatment, and only one recurrence was observed during a median follow-up of 36 months. These findings suggest that EID may be a feasible treatment option for carefully selected patients with rectal GISTs near the DL.

The National Comprehensive Cancer Network guidelines state that neoadjuvant therapy should be considered for locally advanced GISTs in specific anatomical locations, such as the rectum, particularly in cases of genotype-sensitive disease. Although preoperative imatinib treatment can reduce tumor size and increase the likelihood of complete resection, it has several limitations. First, it reduces tumor sizes and mitotic rates, making accurate assessment of recurrence risks difficult [11]. Therefore, preoperative imatinib should be considered only when downstaging the tumor is necessary. Second, there is an ongoing debate regarding its impact on survival outcomes [12,13]. Third, KIT/PDGFRA mutation testing prior to imatinib administration is essential to identify genotypes likely to respond to treatment; preoperative imatinib is not applicable in tumors without sensitive mutations [14]. Fourth, the optimal duration of treatment remains uncertain. While the guidelines recommend treatment for at least 6 months, the exact duration cannot be reliably predicted in individual patients [1]. The current standard treatment for GISTs is surgical resection. However, when GISTs arise in the rectum, surgery often requires general anesthesia and, in many cases, may necessitate sacrificing the anal sphincter, which significantly affects anal function. Although advances in minimally invasive surgery, such as transanal endoscopic microsurgery and transanal minimally invasive surgery, have been made to preserve sphincter function [1,15], these approaches have some limitations. Specifically, they present technical challenges in accessing deep submucosal or muscularis lesions near the DL, limited visualization of tumors in this area, and the potential risk of sphincter damage that can result in reduced anal tone or fecal incontinence [15].

Given these limitations, endoscopic treatment may serve as an effective and less invasive alternative for selected rectal GISTs, particularly those located near the DL. It allows targeted tumor removal while preserving anal sphincter function and minimizing the risk of postoperative complications, offering a function-preserving option in anatomically challenging cases. Although there are currently no large-scale studies on the endoscopic treatment of rectal GISTs, several studies on gastric GISTs have demonstrated the safety and efficacy of endoscopic resection [16,17], suggesting its potential applicability to the lower gastrointestinal tract. In a single-center retrospective study by Marcella et al., 97 patients with upper gastrointestinal GISTs underwent endoscopic resection, including ESD, endoscopic full-thickness resection (EFTR), or submucosal tunneling endoscopic resection. The study reported a 100% en bloc resection rate and a 77.4% complete (R0) resection rate, with no recurrence or metastases observed during a mean follow-up of 21.3 months [17]. Similarly, a Korean study by Kim et al. evaluated 83 gastric GIST cases treated via either ESD or clip-and-cut EFTR, reporting high R0 resection rates with no recurrence during the median follow-up period of 25 months [16]. Despite anatomical differences between the upper and lower GI tract, such findings provide a conceptual foundation for exploring endoscopic treatment as a valid therapeutic approach even in rectal GISTs.

Although several retrospective studies have demonstrated the feasibility of device-assisted EFTR for colorectal subepithelial tumors [18,19], its application near the DL is limited owing to technical challenges, including scope maneuverability, difficulty with defect closure, risk of sphincter injury, and, most importantly, its inapplicability to lesions larger than 2 cm. In contrast, EID offers a more controlled and anatomically favorable approach in this region. Above all, device-assisted EFTR is not covered by the national health insurance system in South Korea and was therefore not available for use in this study. Its reimbursement is currently restricted to the management of iatrogenic perforation, and it is not approved for the elective endoscopic resection of subepithelial tumors. Consequently, EID was the primary endoscopic treatment option available for all eligible patients in this series, regardless of lesion size, when technically feasible based on tumor location and endoscopic findings.

To our knowledge, this is the first study to systematically review and analyze a series of 10 cases of rectal GISTs treated with EID, highlighting its feasibility and clinical value. EID may be a preferred option, especially in these difficult-to-locate cases or in patients with multiple comorbidities, where sphincter preservation is crucial. However, given the small sample size in this study, larger prospective studies are warranted to validate these findings.

In our study, although en bloc resection was achieved in all the patients, the complete resection rate was 70%. All three patients with R1 resection did not have high-risk features and therefore did not receive adjuvant imatinib therapy; to date, none have experienced recurrence. Interestingly, late recurrence was observed in only one case, which involved a high-risk GIST patient who had undergone complete resection. This finding aligns with those of previous studies on gastric GISTs, where the complete resection rate was approximately 80%; however, recurrence remains rare [16,17]. Such discordance may be attributed to the fact that although GISTs are encapsulated and can be easily dissected, their base typically originates from the muscularis propria. During the dissection process, tumor cells may be present within the proper muscle resection margin, where high-frequency cautery was applied, leading to a pathological classification of R1 resection despite apparent gross complete removal. However, these results should be interpreted with caution. Given the small number of patients with R1 resection and the relatively short follow-up period, the favorable outcomes observed in our cohort may not fully reflect the long-term oncologic efficacy of EID. Larger studies with longer follow-up are needed to validate these findings. As endoscopic resection of rectal GISTs is rare, the definition of complete resection in this context has not been firmly established. There is no clear consensus on what constitutes a positive resection margin in this setting, whether it denotes the presence of tumor cells within the diathermy-affected zone or requires a minimum clearance of 1 mm or more from the transected margin. Further studies are warranted to elucidate the prognostic factors and clarify the true clinical significance of pathological resection margins in the endoscopic management of rectal GISTs.

To provide a procedural reference for the feasibility of EID for rectal GISTs near the DL, we compared outcomes with those of ESD performed for epithelial lesions in the same anatomical region. We acknowledge that rectal GISTs (muscularis propria origin) and epithelial lesions (mucosal/submucosal origin) represent different disease entities with inherently different tissue characteristics, which limits direct comparability. However, this comparison was included as an exploratory analysis to provide a practical benchmark for procedural feasibility and safety parameters rather than to compare disease outcomes between different pathologic entities. The comparison was strengthened by age- and sex-matched controls to minimize confounding from patient characteristics. Despite the pathological differences, there were no significant differences in procedure times, en bloc resection rates, complete resection rates, or recurrence rates between the two groups. Adverse events were also not significantly different, indicating that EID can be performed with similar efficacy and safety for rectal GISTs. The similar outcomes between rectal GISTs and epithelial lesions may be explained by the encapsulated nature of GISTs, where dissection typically requires additional time when separating the tumor from its origin layer, that is, the proper muscle. Furthermore, recent advancements in endoscopic equipment—such as improved knives, stabilization devices like the ST hood, and high-performance electrosurgical units—have likely facilitated deep-layer dissection of GISTs. All procedures in this study were performed by expert endoscopists, which likely contributed to the high technical success and safety.

However, performing EIDs near the DL remains technically challenging. The short distal margin, difficulty in achieving an adequate submucosal flap, non-lifting characteristics of some lesions, bleeding tendencies, and limited visualization due to narrow pelvic anatomy all contribute to procedural complexity. These challenges highlight the importance of operator experience, careful device selection, and continuous technical refinements. Nevertheless, further studies are needed to evaluate the learning curve, identify the risk factors for treatment failure, and optimize device utilization.

This study had some limitations. First, this was a small-scale, retrospective, single-center study, which limits the generalizability of the findings. However, this study serves as a valuable feasibility study for exploring the potential of EID for rectal GISTs. Second, KIT/PDGFRA mutation testing was not performed because it is not routinely available in clinical practice in Korea. Third, risk factors associated with treatment failure could not be identified, highlighting the need for larger prospective studies. Fourth, all procedures were performed by expert endoscopists, who may not reflect the outcomes of less experienced surgeons. Fifth, the exact distance from the dentate line was not routinely documented because of the retrospective study design, precluding further analysis of its potential impact on procedural outcomes. Sixth, in addition, defect closure was not standardized in this study; clip closure was applied selectively at the operator’s discretion rather than according to a predefined protocol. This non-standardized, operator-dependent closure strategy may have influenced the observed safety outcomes, including delayed bleeding and perforation. Finally, the follow-up duration, which is sufficient to capture early recurrence, may not be long enough to fully assess long-term outcomes, particularly for slow-growing tumors such as GISTs.

In conclusion, this study reviewed 10 cases of rectal GISTs near the DL treated with EIDs and demonstrated promising outcomes in terms of efficacy, safety, and prognosis. These results support the potential role of EID as a viable treatment modality, particularly in patients in whom surgery is not feasible or who have a high operative risk. Given its technical feasibility and favorable clinical outcomes, EID may be considered a feasible treatment option in carefully selected patients, particularly when preservation of anal sphincter function is important. However, larger prospective studies are warranted to validate these findings.

## Figures and Tables

**Table 1:** Clinicoendoscopic characteristics of patients with rectal gastrointestinal stromal tumors near the dentate line.

	Rectal GIST near the Dentate Line (n = 10)
Age (year, median)	53 (20–71)
Sex (male, n, %)	4 (40.0%)
BMI (kg/m^2^)	26.54 ± 3.71
Smoking (n, %)	1 (10.0%)
Comorbidities (n, %)	
Hypertension	3 (30.0%)
Diabetes mellitus	1 (10.0%)
Chronic kidney disease	0
Cardiovascular disease	1 (10.0%)
ASA class (>3)	0
Anti-thrombotic agent	1 (10.0%)
Bowel preparation (BPPS score)	9
Tumor size (mm, mean ± SD)	25.70 ± 14.09
Growth pattern (endophytic)	10 (100%)
Procedure time (min, mean ± SD)	81.30 ± 46.20
Histopathology *	
Very low risk	4 (40%)
Low risk	5 (50%)
High risk	1 (10%)

GIST: gastrointestinal stromal tumors, BMI: body mass index, ASA: American Society of Anesthesiologists, HPF: high-power field. * Risk was stratified according to the modified National Institutes of Health (Joensuu) consensus criteria, based on tumor size, mitotic count, tumor site, and tumor rupture. As all tumors were located in the rectum, the criteria were applied as follows: very low risk, ≤2 cm and ≤5 mitoses/50 HPF; low risk, >2–5 cm and ≤5/50 HPF; intermediate risk, ≤2 cm and 6–10/50 HPF; high risk, tumor >5 cm with >5/50 HPF, tumor >10 cm, mitotic count >10/50 HPF, tumor rupture, or—given the non-gastric location—tumors >2–5 cm with >5/50 HPF or 5–10 cm with ≤5/50 HPF.

**Table 2:** The efficacy and safety of endoscopic intermuscular dissection for rectal gastrointestinal stromal tumors near the dentate line.

	Rectal GIST near the Dentate Line (n = 10)
Efficacy of EID for rectal GIST	
En bloc resection (n, %)	10 (100%)
Complete resection (n, %)	7 (70%)
Resection speed (mm^2^/min, mean ± SD)	16.91 ± 9.94
Safety of EID for rectal GIST	
Delayed bleeding (n, %)	1 (10.0%)
Perforation (n, %)	1 (10.0%)
PECS (n, %)	1 (10%)
Duration of fasting (days, mean ± SD)	1 (1–3)
Hospital days (days, mean ± SD)	3 (3–8)
* Anorectal dysfunction	0
Recurrence (n, %)	1 (10%)
Follow-up duration (median, min., max., months)	36 (20–66)
Death (n, %)	0

GIST: gastrointestinal stromal tumors, EID: endoscopic intermuscular dissection, PECS: post-endoscopic electrocoagulation syndrome. * Anorectal dysfunction; fecal incontinence, anal injury.

**Table 3:** A summary of clinical characteristics, therapeutic outcomes, and prognosis of all patients with rectal gastrointestinal stromal tumors treated by endoscopic intermuscular dissection.

Case Number	Age/Sex	Procedure Time (min)	En Bloc Resection	Complete Resection	Lesion Size (mm)	Mitosis	Risk Stratification *	Complications	Hospital Days	Prognosis
1	32/F	45	Achieved	Vertical margin(+)	18 × 20	1/50 HPF	Very low risk	None	3	No recurrence
2	36/M	120	Achieved	Achieved	30 × 28	1/50 HPF	Low risk	None	3	No recurrence
3	54/F	58	Achieved	Vertical margin(+)	15 × 12	0/50 HPF	Very low risk	None	3	No recurrence
4	70/M	170	Achieved	Achieved	55 × 40	25/50 HPF	High risk	Delayed perforation Transient fecal urgency	8	Recurrence at the same site at 61 months
5	53/F	24	Achieved	Achieved	7 × 7	0/50 HPF	Very low risk	None	3	No recurrence
6	71/F	72	Achieved	Achieved	42 × 36	0/50 HPF	Low risk	None	3	No recurrence
7	20/M	117	Achieved	Vertical margin(+)	30 × 12	0/50 HPF	Low risk	None	3	No recurrence
8	51/M	107	Achieved	Achieved	19 × 12	4/50 HPF	Low risk	Delayed bleeding	4	No recurrence
9	66/F	50	Achieved	Achieved	19 × 7	0/50 HPF	Very low risk	None	3	No recurrence
10	55/F	60	Achieved	Achieved	22 × 23	0/50 HPF	Low risk	None	3	No recurrence

GIST: gastrointestinal stromal tumor, HPF: high-power field. * Risk was stratified according to the modified National Institutes of Health (Joensuu) consensus criteria, based on tumor size, mitotic count, tumor site, and tumor rupture. As all tumors were located in the rectum, the criteria were applied as follows: very low risk, ≤2 cm and ≤5 mitoses/50 HPF; low risk, >2–5 cm and ≤5/50 HPF; intermediate risk, ≤2 cm and 6–10/50 HPF; high risk, tumor >5 cm with >5/50 HPF, tumor >10 cm, mitotic count >10/50 HPF, tumor rupture, or—given the non-gastric location—tumors >2–5 cm with >5/50 HPF or 5–10 cm with ≤5/50 HPF.

**Table 4:** Comparison with endoscopic submucosal dissection for rectal epithelial lesions near the dentate line ^#^.

	Rectal GIST (n = 10)	Epithelial Lesion (n = 20)	*p*-Value
Age (year, median)	53 (20–71)	64 (35–82)	0.099
Sex (male, n, %)	4 (40.0%)	14 (70.0%)	0.139
BMI (kg/m^2^)	26.54 ± 3.71	25.36 ± 2.58	0.159
Smoking (n, %)	1 (10.0%)	4 (20.0%)	0.640
Comorbidities (n, %)			
Hypertension	3 (30.0%)	8 (40.0%)	0.702
Diabetes mellitus	1 (10.0%)	4 (20.0%)	0.640
Chronic kidney disease	0	2 (10.0%)	0.540
Cardiovascular disease	1 (10.0%)	5 (25.0%)	0.633
ASA class (>3)	0	5 (25.0%)	0.140
Anti-thrombotic agent	1 (10.0%)	5 (25.0%)	0.633
Bowel preparation (BPPS)	9	9	
Tumor size (mm, mean ± SD)	25.70 ± 14.09	22.80 ± 12.46	0.551
Procedure time (min, mean ± SD)	81.30 ± 46.20	76.50 ± 32.42	0.758
Histopathology (n, %)			
Benign neoplasm		8 (40.0%)	
Advanced neoplasm		9 (45.0%)	
Adenocarcinoma		3 (15.0%)	
GIST	10 (100%)		
Efficacy			
En bloc resection (n, %)	10 (100%)	20 (100%)	1.000
Complete resection (n, %)	7 (70%)	19 (95.0%)	0.095
Resection speed (mm^2^/min, mean ± SD)	16.91 ± 9.94	12.98 ± 11.95	0.169
Safety			
Delayed bleeding (n, %)	1 (10.0%)	2 (10%)	1.000
Perforation (n, %)	1 (10.0%)	0	0.333
PECS (n, %)	1 (10%)	4 (20%)	0.640
Duration of fasting (days, median)	1 (1–3)	1 (1–2)	0.587
Hospital days (days, median)	3 (3–8)	3 (3–8)	0.865
Recurrence (n, %)	1 (10%)	0	0.333
Follow-up duration (median, min., max., months)	36 (20–66)	30 (12–125)	0.619
Death (n, %)	0	0	1.000

GIST: gastrointestinal stromal tumors, BMI: body mass index, ASA: American Society of Anesthesiologists, EID: endoscopic intermuscular dissection, PECS: post-endoscopic electrocoagulation syndrome. ^#^ Owing to the small sample size and low event rate, this study was underpowered to detect statistically significant differences between the two groups. Non-significant *p*-values should therefore not be interpreted as evidence of equivalent outcomes, and all comparisons should be regarded as exploratory.

## Data Availability

The data presented in this study are available upon request from the corresponding author.

## Supplementary Materials

The following supporting information can be downloaded at: https://www.mdpi.com/article/10.3390/jcm15145712/s1.
